# The effect of new atypical antipsychotic drugs on the expression of transcription factors regulating cytochrome P450 enzymes in rat liver

**DOI:** 10.1007/s43440-024-00608-2

**Published:** 2024-06-15

**Authors:** Przemysław J. Danek, Władysława A. Daniel

**Affiliations:** Department of Pharmacokinetics and Drug Metabolism, Maj Institute of Pharmacology, Polish Academy of Sciences, Smętna 12, 31-343 Kraków, Poland

**Keywords:** Cytochrome P450, Atypical neuroleptics, Chronic treatment, Rat liver, Transcription factors

## Abstract

**Background:**

Our recent studies showed that prolonged administration of novel atypical antipsychotics affected the expression and activity of cytochrome P450 (CYP), as demonstrated in vitro on human hepatocytes and in vivo on the rat liver. The aim of the present work was to study the effect of repeated treatment with asenapine, iloperidone, and lurasidone on the expression of transcription factors regulating CYP drug-metabolizing enzymes in rat liver.

**Methods:**

The hepatic mRNA (qRT-PCR) and protein levels (Western blotting) of aryl hydrocarbon receptor (AhR), pregnane X receptor (PXR), constitutive androstane receptor (CAR) and peroxisome proliferator-activated receptor (PPARγ) were measured in male Wistar rats after 2 week-treatment with asenapine, iloperidone or lurasidone.

**Results:**

The 2-week treatment with asenapine significantly diminished the AhR and PXR expression (mRNA, protein level), and CAR mRNA level in rat liver. Iloperidone lowered the AhR and CAR expression and PXR protein level. Lurasidone did not affect the expression of AhR and CAR, but increased PXR expression. The antipsychotics did not affect PPARγ.

**Conclusions:**

Prolonged treatment with asenapine, iloperidone, or lurasidone affects the expression of transcription factors regulating the CYP drug-metabolizing enzymes. The changes in the expression of AhR, CAR, and PXR mostly correlate with alterations in the expression and activity of respective CYP enzymes found in our previous studies. Since these transcription factors are also engaged in the expression of phase II drug metabolism and drug transporters, changes in their expression may affect the metabolism of endogenous substrates and pharmacokinetics of concomitantly used drugs.

**Supplementary Information:**

The online version contains supplementary material available at 10.1007/s43440-024-00608-2.

## Introduction

Antipsychotic drugs are substrates and inhibitors of cytochrome P450 (CYP) enzymes including CYP2D6, CYP3A4 and CYP2C (reviewed in [1, 2]). Phenothiazines, haloperidol or older atypical antipsychotics, like olanzapine, directly inhibit (via binding to the enzyme protein) the activity of CYP2D6, and with a lower potency, of CYP1A2, CYP2C9, and CYP3A4 [1, 3, 4]. Our recent studies have shown that the novel atypical neuroleptics can also directly inhibit CYP enzymes: asenapine is an inhibitor of CYP1A2 and CYP2D6, iloperidone suppresses CYP3A4, CYP2C19, and CYP2D6, while lurasidone moderately inhibits CYP1A2, CYP2C9 and CYP219 [5, 6].

After prolonged treatment, antipsychotics can affect cytochrome P450 enzymes’ expression and activity, which has been shown in vitro on human hepatocytes and in vivo on the rat liver (Table 1). Specifically, when added to the cell culture for 5 days, levomepromazine and clozapine enhanced the mRNA level and activity of CYP3A4, not affecting CYP1A1/2, CYP2C9 and CYP2C19 [7]. In contrast, iloperidone diminished the mRNA level and activity of CYP3A4, asenapine diminished those of CYP1A2, while lurasidone did not affect the investigated CYP enzymes [8].

**Table 1:**
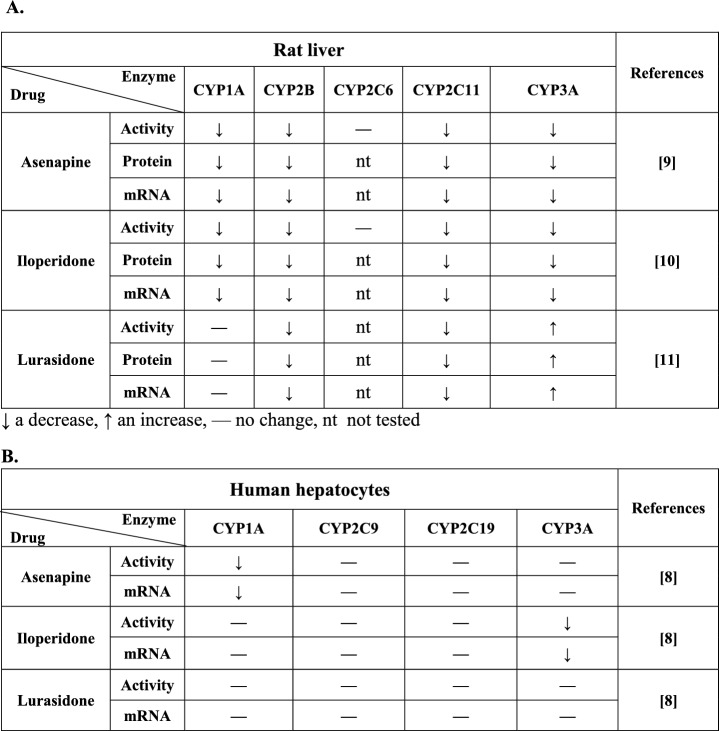
The effect of asenapine, iloperidone and lurasidone on the mRNA, protein level and activity of cytochrome P450 enzymes in the liver of male rats treated with one of the antipsychotics for two weeks (A), and in human hepatocyte culture after 5 day-exposure to one of the antipsychotics (B)

↓ a decrease, ↑ an increase, — no change, nt not tested

When given to rats for two weeks, the atypical neuroleptics: asenapine and iloperidone reduced the expression and activity of CYP1A, CYP2B, CYP2C11, and CYP3A enzymes in the liver, not affecting the CYP2C6 enzyme [9, 10]. On the other hand, lurasidone did not affect human CYP enzymes in hepatocyte culture, but decreased the expression of CYP2B1/2 and CYP2C11, and enhanced that of CYP3A1/2 in rat liver after chronic treatment [11].

The above-presented data indicate similarities in the downregulation of CYP1A by asenapine and of CYP3A by iloperidone in humans and rats. However, the difference in the effects of lurasidone between in vitro experiments on human hepatocyte culture and in vivo experiments performed in rats has been noticed, namely, lurasidone enhanced the CYP3A expression in rat liver, but not in human hepatocyte culture. Although there are similarities in the expression regulation of CYP enzymes in humans and rats, some differences between the two species have also been noticed, e.g. in the induction of CYP3A subfamily enzymes by rifampicin and dexamethasone [12, 13]. Moreover, in vivo*,* additional mechanisms are involved in CYP enzyme regulation by neuroactive drugs, including neuroendocrine and neuroimmune pathways [14–16].

Taking into account the contribution of cytoplasmic aryl hydrocarbon receptor (AhR) to the regulation of *CYP1A* expression and the involvement of nuclear receptors: pregnane X receptor (PXR), constitutive androstane receptor (CAR) and retinoid X receptor (RXR) in the expression of *CYP3A* and other *CYP* genes in humans and rodents [17–20] it can be expected that asenapine interacts with human and rat AhR, iloperidone interplays with PXR and/or CAR, while a possibility of cooperation of lurasidone with the mentioned *CYP* transcription factors is difficult to assess at this stage of studies. Thus, the molecular mechanisms of CYP enzyme regulation by antipsychotics in the liver need further investigation.

The present study aimed to identify the effect of 2 week-treatment treatment with the new atypical antipsychotic drugs: asenapine, iloperidone, and lurasidone on the expression of transcription factors regulating the CYP drug-metabolizing enzymes in rat liver.

## Materials and methods

### Animals

The experiments were conducted on male Wistar Han rats (3 months old and weighing 280–300 g) from Charles River Laboratories, Sulzfeld, Germany. The rats were housed under standard laboratory conditions (22 ± 2 °C room temperature, 50 ± 5% room humidity, and 12:12 light: dark cycle) with free access to water and food. All animal procedures and experiments were conducted in strict accordance with the European regulations (EU Directive 2010/63/EU), and approved by the Local Ethics Commission for Experimentation on Animals at the Maj Institute of Pharmacology, Polish Academy of Sciences, Kraków.

### Experimental design

Rats (*n* = 12 for each group) received an antipsychotic drug or vehicle (control) once daily for two weeks. Asenapine (0.3 mg/kg, Sigma-Aldrich, St. Louis, MO, USA; product number A7861) dissolved in saline (vehicle) was administrated subcutaneously (sc). Iloperidone (1 mg/kg, TargetMol, Boston, MA, USA; product number T1539) dissolved in 1% Tween 80 in sterile water (vehicle) or lurasidone (1 mg/kg, TargetMol, Boston, MA, USA; product number T1735) dissolved in 0.5% methylcellulose and 0.2% Tween 80 (vehicle) were injected intraperitoneally (ip). The doses of drugs used in this experiment were selected based on our previous studies [9–11]. All animals were sacrificed by decapitation 24 h after the final drug/vehicle injection, and the whole livers were removed, frozen in dry ice, and stored at − 80 °C.

### Determination of total protein in the liver

The frozen livers (20 mg) were homogenized (TissueLyser, Qiagen, Germany) in ice-cold Tissue Extraction Reagent I (Thermo Fisher Scientific, Walthman, MA, USA) supplemented with 1 mM PMSF (Sigma, Sigma-Aldrich, St. Louis, MO, USA; product number P7626). Then, the homogenates were centrifuged for 15 min at 15,000 × g at 4 °C, and supernatants were collected. The total protein content in the sample was assessed by Pierce BCA Protein Assay Kit (Thermo Fisher Scientific, Walthman, MA, USA; product number 23225).

### Western blot analyzes

Western blot was performed as described previously by Danek et al. [9, 10] with some modifications. Briefly, samples (30 μg protein) were separated on a 12% or 8% (for AhR) SDS-PAGE gel in a Laemmli buffer system (BioRad, Hercules, CA, USA; product number #1,610,737), and electrotransferred (100 V, 90 min) onto a nitrocellulose membrane (Merck KGaA, Darmstadt, Germany). Then, the membranes were blocked with 5% nonfat milk in Tris-buffered saline with 0.1% Tween 20 overnight at 4 °C. The membranes were probed with diluted primary antibodies as follows: rabbit anti-MB67 (CAR) (1:1000, Thermo Fisher Scientific, Walthman, MA, USA; product number PA5-36,268), rabbit anti-PPARγ (1:1000, Thermo Fisher Scientific, Walthman, MA, USA; product number PA3-821A), rabbit anti-PXR (1:1000, Thermo Fisher Scientific, Walthman, MA, USA; product number PA5-116,715), mouse anti-AHR (1:1000, Thermo Fisher Scientific, Walthman, MA, USA; product number MA1-514) and mouse anti-β-actin (1:15,000, Sigma-Aldrich, St. Louis, MO, USA; product number: A1978). Then, the blots were incubated with the appropriate (anti-mouse IgG or anti-rabbit IgG) horseradish peroxidase-conjugated secondary antibodies (Vector Laboratories, Burlingame, CA, USA; product number: PI-1000; Jackson ImmunoResearch, West Grove, PA, USA; product number: 115-035-003, respectively). The bands were evaluated using a luminescent image analyzer (LAS-1000, Fuji-film, Tokio, Japan), and relative levels of immunoreactivity were quantified using the Image Gauge software (Fujifilm, Tokio, Japan). The data were normalized to protein level based on the β-actin levels (all original immunoblots generated during the study are included in Supplementary Materials, Fig. S1).

### Gene expression

The RT–qPCR method conducted in this study has been previously described by Danek et al. [11]. In brief, the liver tissue was dissected, quickly frozen in dry ice, and stored at − 80 °C until analysis. Total RNA was isolated using the Total RNA Mini kit (A&A Biotechnology, Gdynia, Poland; product number: 031–100). The total RNA concentration was measured using a Synergy/HTX multi-mode reader (BioTek, Winooski, VT, USA). The extracted RNA was reversely transcribed using a High-Capacity cDNA Reverse Transcription Kit (Thermo Fisher Scientific, Walthman, MA, USA; product number: 4368814). Quantitative real-time PCR was performed in duplicate using TaqMan® Gene Expression Assays [*Nr1i2* (PXR)—Rn00583887_m1; *Nr1i3* (CAR)—Rn000576085_m1; *Ahr*- Rn00565750_m1; *PPARγ*—Rn00440945_m1 and *Actb*—Rn00667869_m1, Thermo Fisher Scientific, Walthman, MA, USA) with TaqMan™ Universal Master Mix (Thermo Fisher Scientific, Walthman, MA, USA; product number: 4369016) and the BioRad CFX96 PCR system (BioRad, Hercules, CA, USA). The abundance of RNA was calculated according to the following equation: abundance = 2^−(threshold cycle)^. The results were normalized to the *Actb* (*β-actin*) gene.

### Data analysis

The antipsychotic drugs asenapine, iloperidone, and lurasidone were investigated in separate experiments. Therefore, the results of each experiment were calculated and statistically analyzed separately. The Kolmogorov–Smirnov test was used to assess normality. Changes in the mRNA and protein expression levels of transcription factors were statistically assessed using an unpaired Student’s t-test (GraphPad Prism 10 Software, Inc., La Jolla, CA). The obtained values were indicated as the mean ± SD and were recognized as significant when p ≤ 0.05.

## Results and discussion

This is the first report showing that prolonged treatment with the new atypical antipsychotic drugs: asenapine, iloperidone, and lurasidone affects the expression of transcription factors regulating the cytochrome P450 drug-metabolizing enzymes. The observed changes in the expression of transcription factors AhR, CAR, and PXR mostly correlate with the alterations in the expression and activity of respective CYP enzymes found in our previous studies in rats [9–11] and humans [8], after prolonged exposure to the antipsychotics (Table 1).

**Fig. 1:**
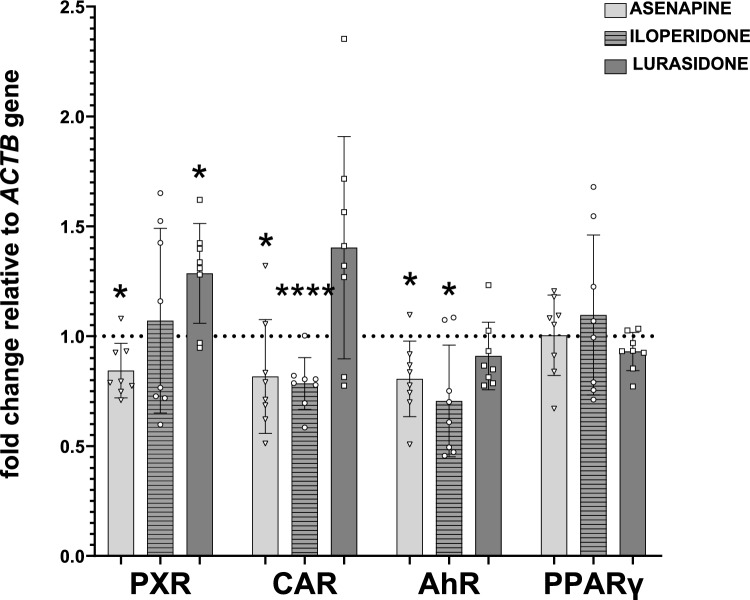
The effect of asenapine (0.3 mg/kg sc), iloperidone (1 mg/kg ip), and lurasidone (1 mg/kg ip) on the mRNA level of the transcription factors PXR, CAR, AhR, and PPARγ in rat liver, after 2 week-treatment with one of the antipsychotics. All values are shown as the mean ± SD (*n* = 8). The results were evaluated statistically using Student’s *t*-test: *p < 0.05; ****p < 0.0001 versus control group (asenapine PXR *t*_14_ = 2.164, *p* = 0.048; lurasidone PXR *t*_14_ = 2.438, p = 0.0287; aseanpine CAR *t*_14_ = 2.299, *p* = 0.0374; iloperidone CAR *t*_14_ = 23.79, *p* < 0.0001; asenapine AHR *t*_14_ = 2.171, *p* = 0.0476; iloperidone AhR *t*_14_ = 2.261, *p* = 0.0402)

**Fig. 2:**
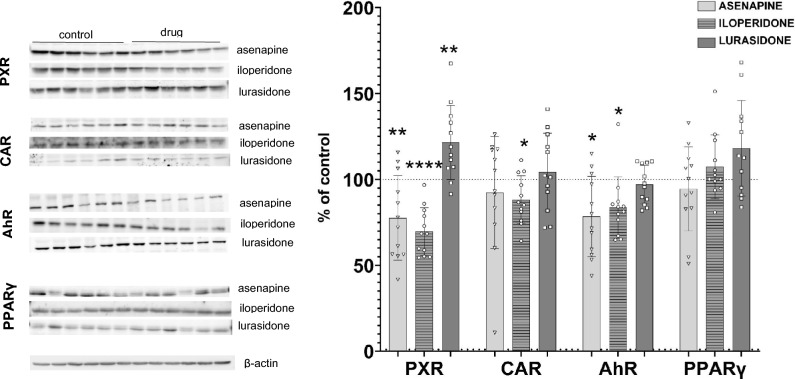
The effect of asenapine (0.3 mg/kg sc), iloperidone (1 mg/kg ip), and lurasidone (1 mg/kg ip) on the protein level of the transcription factors PXR, CAR, AhR, and PPARγ in rat liver, after 2 week-treatment with one of the antipsychotics. All values are shown as the mean ± SD (*n* = 12). The results were evaluated statistically using Student’s *t*-test: *p < 0.05; **p < 0.01; ****p < 0.0001 versus control group (asenapine PXR *t*_22_ = 2.831, *p* = 0.0097; iloperidone PXR *t*_22_ = 5.013, *p* < 0.0001; lurasidone PXR *t*_22_ = 2.847, *p* = 0.0094; iloperidone CAR *t*_22_ = 2.571, *p* = 0.0174; asenapine AHR *t*_22_ = 2.722, *p* = 0.0125; iloperidone AhR *t*_22_ = 2.310, *p* = 0.0306)

The two-week treatment with asenapine significantly decreased the expression (mRNA and protein level) of AhR (Figs. 1, 2) in rat liver, which corresponded with the reduction in the CYP1A2 expression and activity after two-week treatment of rats with this antipsychotic drug, observed in previous experiments [9]. Simultaneously, asenapine decreased PXR expression and CAR mRNA level, which, in turn, was consistent with the depletion of CYP2B1 and CYP3A1 expression and activity found earlier in those animals (Figs. 1, 2). Both transcription factors are engaged in the regulation of those two CYP subfamily enzymes to different degrees. CAR is the main regulator of CYP2Bs, while PXR governs the expression of CYP3As [19]. The above results concerning the effect of asenapine on rat AhR and CYP1A2 enzyme in vivo remain in agreement with the results obtained for the corresponding CYP1A2 enzyme in humans in vitro. Just as in rats in vivo, asenapine decreased the *CYP1A2* mRNA and activity in humans in vitro after 5 day-incubation with hepatocyte culture [8] (Table 1).

Prolonged administration of iloperidone exerted similar effects on the expression of the investigated transcription factors in rat liver. Iloperidone lowered the expression of AhR (mRNA and protein level) (Figs. 1, 2), which was consistent with the reduced CYP1A expression and activity observed after prolonged antipsychotic treatment in our previous experiment [10]. However, iloperidone did not influence the *CYP1A2* expression in human hepatocytes, which may suggest different amino acid sequence in the ligand binding domain of AhR receptor between species [8, 21]. At the same time, iloperidone diminished CAR expression and PXR protein level, but not PXR mRNA level (Figs. 1, 2). The observed discrepancy between PXR mRNA and protein level may result from the decreased stability of PXR protein. The revealed decreases in the level of CAR and PXR proteins evoked by iloperidone remain in agreement with the decrease in the expression and activity of CYP2B1/2 and CYP3A1/2 enzymes in the liver of chronically treated rats, detected in our earlier studies [10]. In this case, the results obtained in rats correlated positively with those concerning changes in the CYP3A4 mRNA and activity produced by iloperidone in human hepatocyte culture [8] (Table 1).

In contrast to asenapine and iloperidone, lurasidone did not affect the expression of AhR and CAR but increased the expression of PXR (mRNA and protein level) (Figs. 1, 2), which corresponded with the lack of lurasidone effect on CYP1A expression and activity, and enhanced expression and activity of CYP3A1/2 (but not CYP2B) enzymes found in our previous study in those animals [11]. Since PXR is not the main regulator of CYP2B enzymes and the CAR expression was not changed by lurasidone, the observed reduction in the CYP2B expression in the rat may be ascribed to changes in hormone levels (decreased growth hormone and corticosterone, and increased T_3_ level), as observed for other antipsychotics in rats in vivo [9, 10]. But the lack of effect of lurasidone on CYP3A4 noticed in human hepatocyte culture [7] compared to the increase in the CYP3A1/2 [11] and PXR expression observed in the rat liver in vivo may be explained by structural differences in the *CYP3A* genes’ responsive elements or PXR protein, which leads to disparate regulation of CYP3A enzymes in humans and rodents [19, 22]. The above discrepancy in the regulation of CYP3A expression by lurasidone may also be related to structural and functional differences between human and rat glucocorticoid receptor (GR), which takes part in the regulation of PXR and CAR expression [18]. Thus, rifampicin is a strong PXR and CYP3A4 inducer in humans, dexamethasone is a much more efficient inducer of those proteins in rats than in humans, while pregnenolone 16α-carbonitrile has been found to be a specific PXR ligand and CYP3A inducer in rats [12, 23, 24]. On the other hand, other mechanisms may also be engaged in the regulation of cytochrome P450 by neuroactive drugs in vivo*,* which cannot be seen in hepatocyte culture, such as the presence of high concentrations of drug metabolites, which may exert different effects on cytochrome P450 expression than their parent compounds.

The investigated antipsychotics did not affect the expression of the transcription factor PPARγ, which is involved in the regulation of hepatic lipid metabolism and expression of CYP2D enzymes in the brain [25, 26]. However, it cannot be excluded that some of the tested drugs may act as direct ligands of cytoplasmic/nuclear receptors regulating *CYP* genes [25], which requires further molecular studies. Such a possibility was suggested for the typical antipsychotic chlorpromazine in relation to CAR [18, 28].

In summary, the results obtained in rats indicate that the atypical antipsychotics: asenapine, iloperidone, and lurasidone influence the expression of the transcription factors AhR, PXR, and CAR engaged in the regulation of drug-metabolizing enzymes. Further studies are advisable to find out whether similar changes in the expression of transcription factors are evoked by the tested antipsychotics in humans. The issue seems to be of high importance, since these transcription factors are involved in the expression of enzymes catalyzing phase I drug metabolism (CYP1-3 enzymes) and phase II drug biotransformation (UDP-glucuronosyltransferase, sulfotransferase, glutathione S-transferase), and in the expression of drug transporters (P-glycoprotein, MRPs, OATP2) (reviewed in [29]). Thus, the investigated antipsychotics may affect the pharmacokinetics of concurrently administered drugs. Moreover, some endogenous, physiologically important substances are substrates for phase I and/or phase II drug-metabolizing enzymes (e.g. steroid hormones, cholesterol derivatives, bile acids, thyroid hormones) (discussed in [30]). Therefore, changes in the expression level of the investigated transcription factors may contribute to potential adverse effects of those antipsychotic drugs and drug-drug interactions.

### Supplementary Information

Below is the link to the electronic supplementary material.

Supplementary file1 (PDF 1156 KB)

## Data Availability

The datasets are available from the corresponding author upon reasonable request. All immunoblots generated during the study are included in Supplementary Materials.

## Abbreviations

AhR: Aryl hydrocarbon receptor
CAR: Constitutive androstane receptor
CYP: Cytochrome P450
HPLC: High-performance liquid chromatography
MRP: Multidrug resistance protein
OATP2: Organic anion transporter 2
PPAR: Peroxisome proliferator-activated receptor
PXR: Pregnane X receptor
