# Supplementary material for: The effect of new atypical antipsychotic drugs on the expression of transcription factors regulating cytochrome P450 enzymes in rat liver
Source: Pharmacol Rep. 2024 Jun 15;76(4):895–901. doi: 10.1007/s43440-024-00608-2 (PMC11294401; doi:10.1007/s43440-024-00608-2)

Fig. S1

A.

PXR – 50 kDa

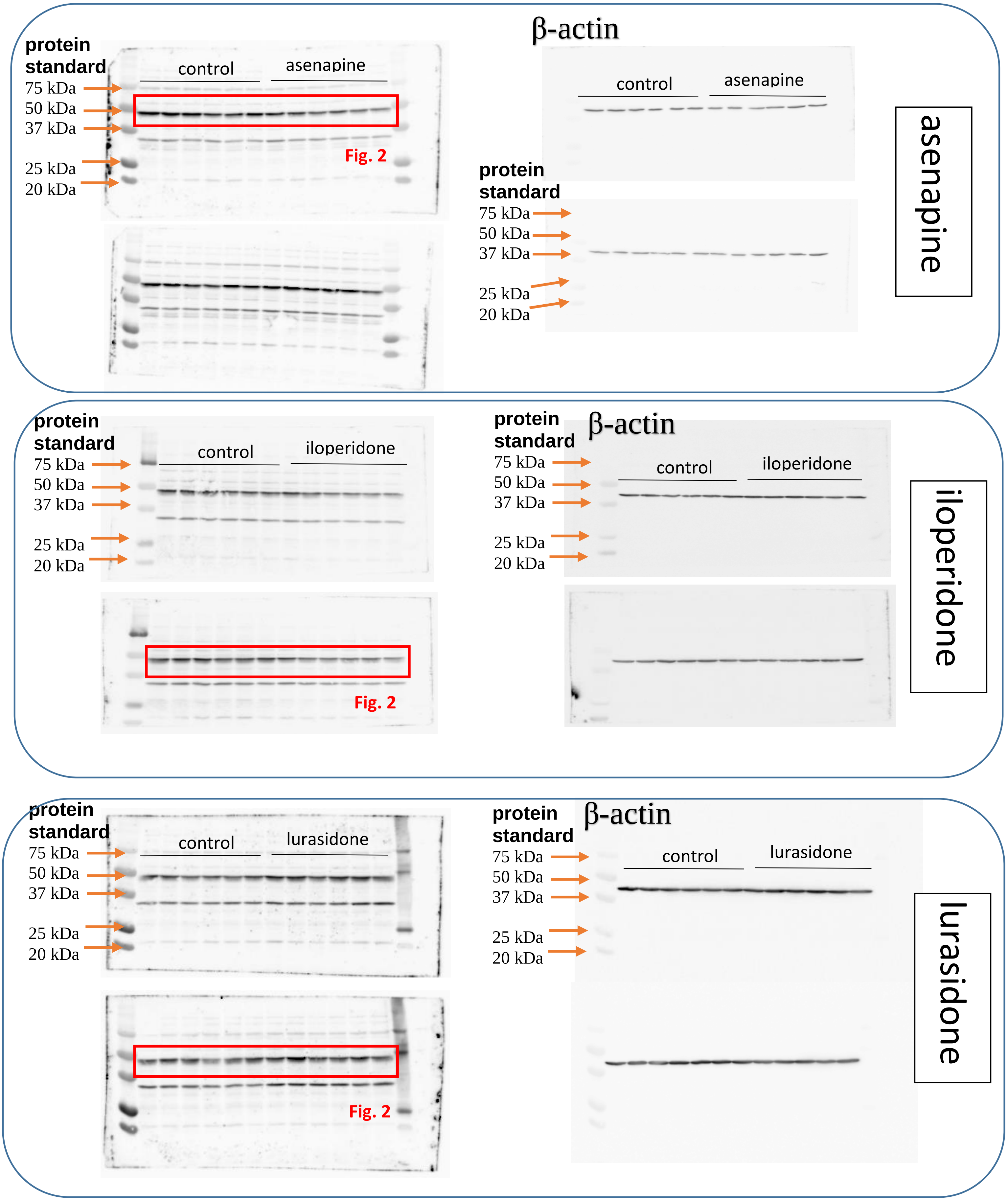

B. CAR- 35 kDa

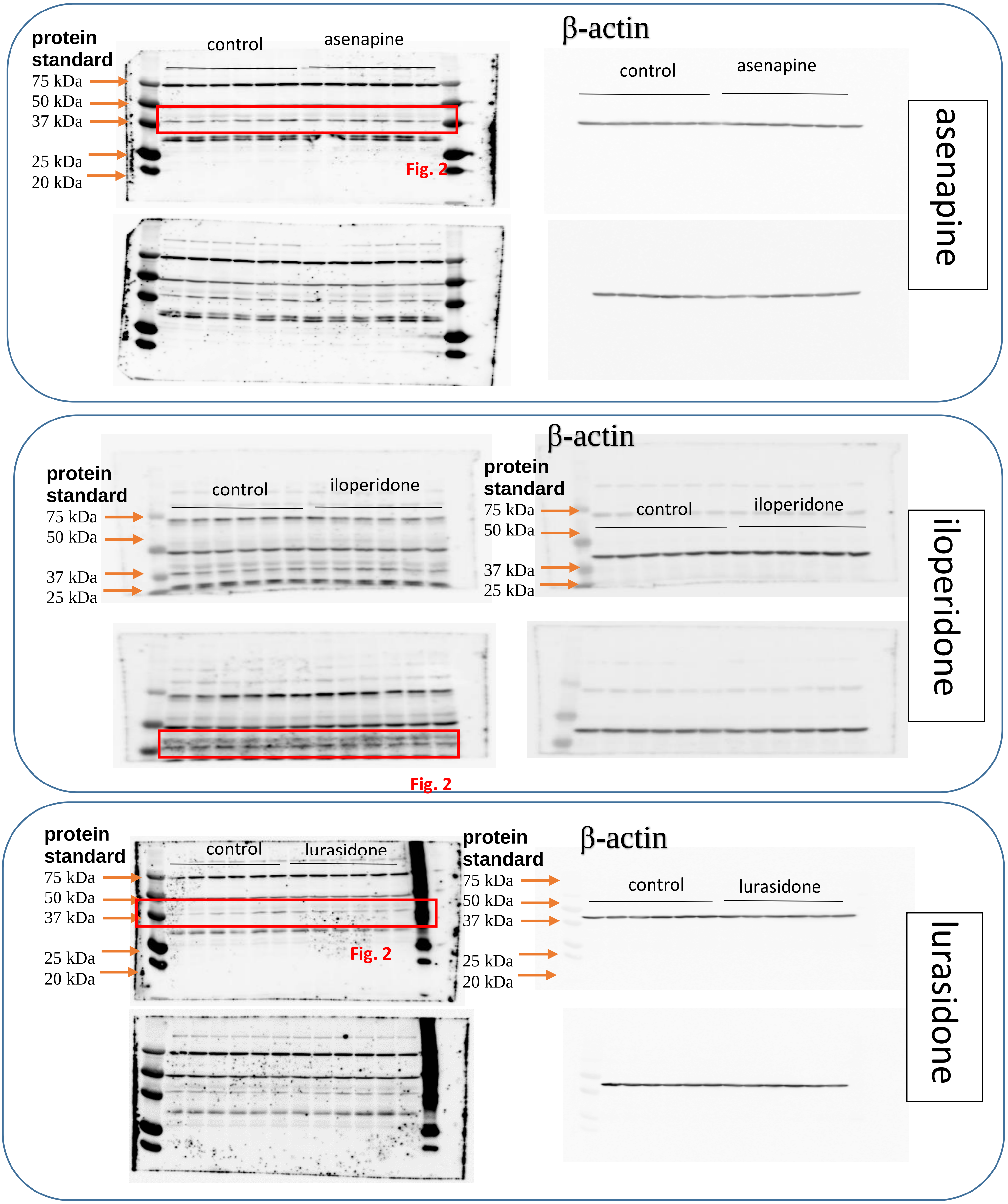

protein standard
control
asenapine
75 kDa
50 kDa
37 kDa
25 kDa
20 kDa
Fig. 2
β-actin
control
asenapine
asenapine
protein standard
control
iloperidone
75 kDa
50 kDa
37 kDa
25 kDa
β-actin
protein standard
75 kDa
50 kDa
37 kDa
25 kDa
control
iloperidone
iloperidone
Fig. 2
protein standard
control
lurasidone
75 kDa
50 kDa
37 kDa
25 kDa
20 kDa
Fig. 2
protein standard
75 kDa
50 kDa
37 kDa
25 kDa
20 kDa
β-actin
control
lurasidone
lurasidone

C.

AhR – 100 kDa

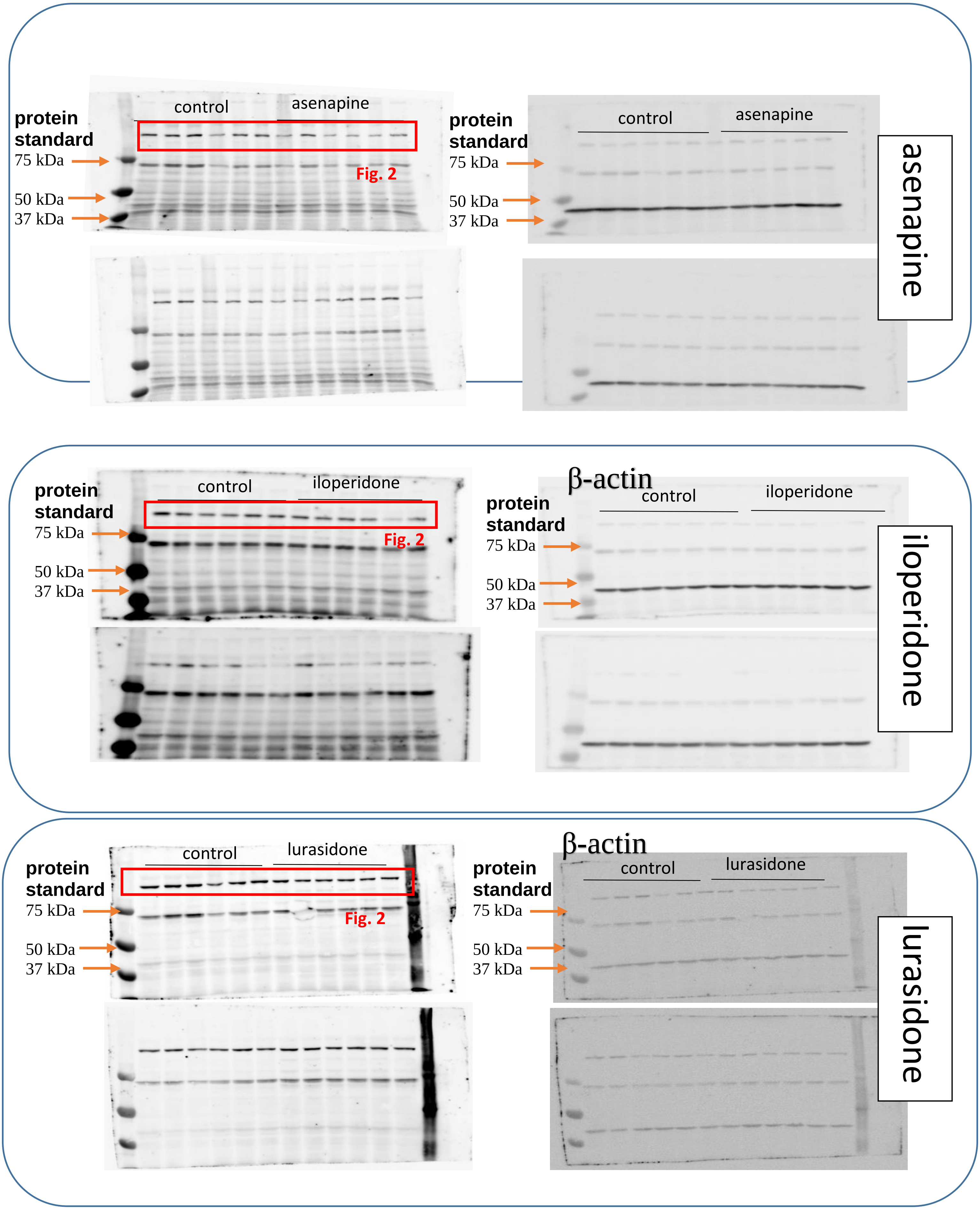
protein standard
75 kDa
50 kDa
37 kDa
control
asenapine
Fig. 2
protein standard
75 kDa
50 kDa
37 kDa
control
asenapine
asenapine
protein standard
75 kDa
50 kDa
37 kDa
control
iloperidone
Fig. 2
β-actin
protein standard
75 kDa
50 kDa
37 kDa
control
iloperidone
iloperidone
protein standard
75 kDa
50 kDa
37 kDa
control
lurasidone
Fig. 2
β-actin
protein standard
75 kDa
50 kDa
37 kDa
control
lurasidone
lurasidone

D. PPARγ- 52+55 kDa

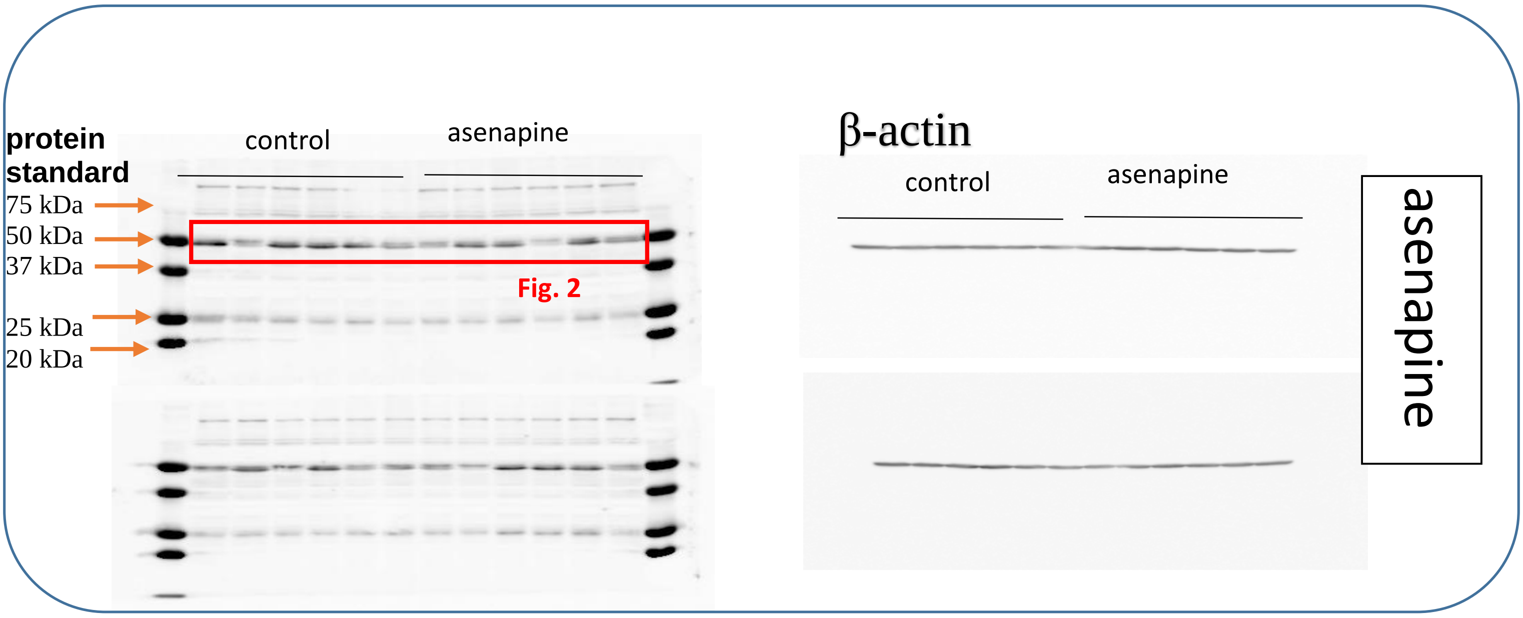

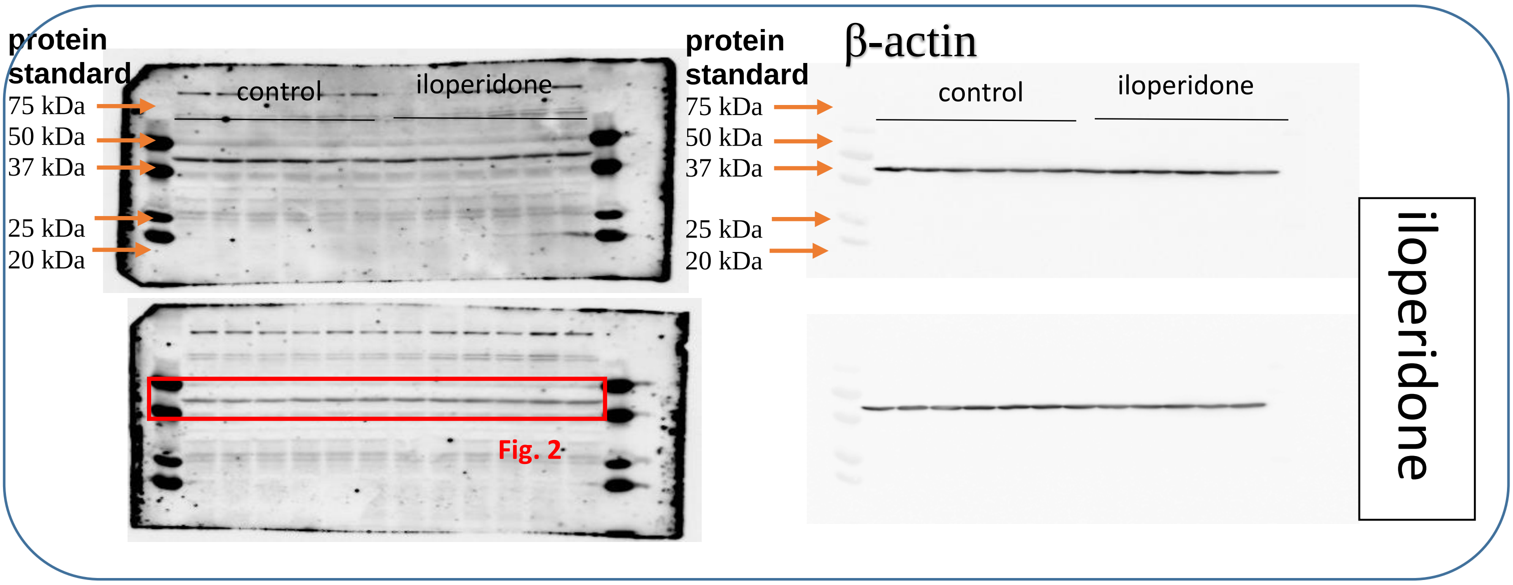

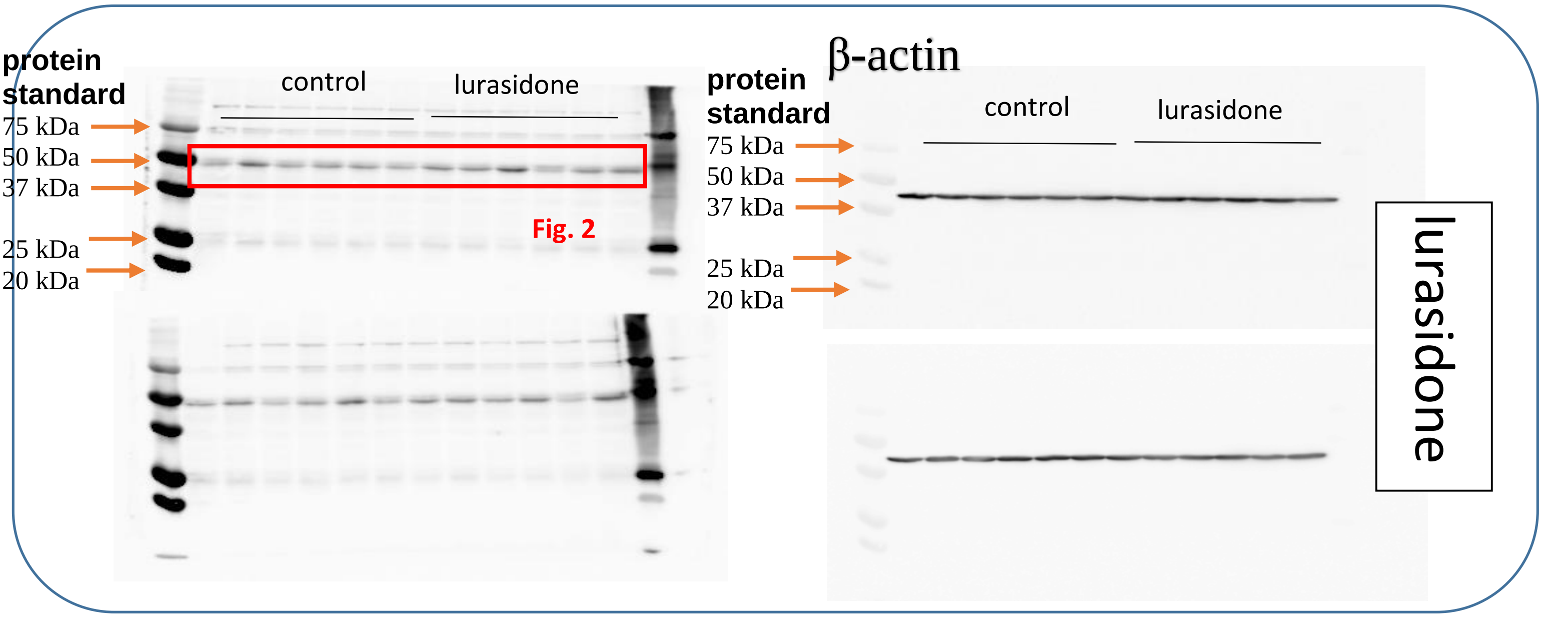

Supplement: Supplementary file 1 — Supplementary file1 (PDF 1156 KB) [file 43440_2024_608_MOESM1_ESM.pdf]
